# Diagnostic agreement between emergency medical service and emergency department physicians, a prospective multicentre study

**DOI:** 10.1186/s12873-024-01041-7

**Published:** 2024-07-18

**Authors:** Lars I. Veldhuis, P. Gouma, Prabath W. B. Nanayakkara, J. Ludikhuize

**Affiliations:** 1 Emergency Department, Amsterdam University Medical Centres, location Academic Medical Centre, Amsterdam, The Netherlands; 2 Department of Anaesthesiology, Erasmus Medical Center, Rotterdam, The Netherlands; 3 Department of Internal Medicine, Amsterdam Public Health Research Institute, Amsterdam University Medical Centres, location VU University Medical Centre, Amsterdam, The Netherlands; 4 Intensive Care Unit, Haga Hospital, The Hague, The Netherlands

**Keywords:** Hospital admission, Deterioration, Emergency department, Early warning score, Emergency Medicine Services, Diagnostic accuracy

## Abstract

**Introduction:**

Early and adequate preliminary diagnosis reduce emergency department (ED) and hospital stay and may reduce mortality. Several studies demonstrated adequate preliminary diagnosis as stated by emergency medical services (EMS) ranging between 61 and 77%. Dutch EMS are highly trained, but performance of stating adequate preliminary diagnosis remains unknown.

**Methods:**

This prospective observational study included 781 patients (> 18years), who arrived in the emergency department (ED) by ambulance in two academic hospitals. For each patient, the diagnosis as stated by EMS and the ED physician was obtained and compared. Diagnosis was categorized based on the International Classification of Diseases, 11th Revision.

**Results:**

The overall diagnostic agreement was 79% [95%-CI: 76–82%]. Agreement was high for traumatic injuries (94%), neurological emergencies (90%), infectious diseases (84%), cardiovascular (78%), moderate for mental and drug related (71%), gastrointestinal (70%), and low for endocrine and metabolic (50%), and acute internal emergencies (41%). There is no correlation between 28-day mortality, the need for ICU admission or the need for hospital admission with an adequate preliminary diagnosis.

**Conclusion:**

In the Netherlands, the extent of agreement between EMS diagnosis and ED discharge diagnosis varies between categories. Accuracy is high in diseases with specific observations, e.g., neurological failure, detectable injuries, and electrocardiographic abnormalities. Further studies should use these findings to improve patient outcome.

## Introduction

The emergency medical services (EMS) and emergency department (ED) staff encounter diverse disease presentations. Admissions to hospital are primarily through the ED and therefore reflects an important entry point into the acute care chain with the hospital environment. It is known that the correct (suspected) diagnosis by EMS personnel can speed up the diagnostic and therapeutic processes at the ED. [1] On the other hand, an incorrect preliminary diagnosis from EMS can have negative effects on patient outcomes, leading to increased frequency of intensive care unit (ICU) admissions, prolonged ED and hospital stays, as well as higher mortality rates. [2–4] Therefore, the preliminary diagnosis by EMS should be correct.

Previous studies examined the accuracy of preliminary diagnoses made by EMS compared to the discharge diagnoses in the ED. These studies have reported overall accuracies ranging from 61 to 77%. [5, 6] However, the accuracy varied depending on the type of disease. [5–8] For instance, Koivulahti et al. reported rates of agreement in categories such as mental health and intoxication (86%), cerebral strokes (81%), respiratory emergencies (58%), and infectious diseases (31%). [5] Similar levels of agreement have been documented for respiratory emergencies in other studies. [7] However, some categories, such as cerebral strokes and mental health problems, have variable outcomes in terms of diagnostic accuracy in comparable research. [6]

Focused training has been shown to improve the accuracy of EMS in stating adequate diagnosis. [5] In the Netherlands, EMS nurses receive extensive training and are probably among the most highly trained in the world. Given the pivotal role of preliminary diagnosis in patient outcomes, it is important to ensure its validity within the Dutch context. Therefore, our study aims to investigate the accuracy of EMS (preliminary) compared to the ED diagnosis with two large Dutch university hospitals. The outcomes were also correlated to relevant clinical outcomes.

## Methods

### Study design and setting

This prospective observational study was performed at both locations of the Amsterdam University Medical Centre. From March 11th until October 28th, 2021, all adult patients (> 18years) presented to the ED by ambulance were included. Excluded patients were those with ongoing cardiopulmonary resuscitation and inter-hospital transfers.

### Dutch EMS system

In the Netherlands, each ambulance team consist of an EMS nurse and a driver. EMS nurses hold licenses to administer Advanced Life Support and have completed a foundational nursing program, complemented by specialized training in areas such as emergency medicine, intensive care medicine, or cardiac care medicine, prior to embarking on their EMS traineeship. EMS drivers, on the other hand, undergo training to provide medical support to EMS nurses and to ensure the safe transportation of patients via ambulance.

### Ethical concerns

Ethical approval was received by the Medical Ethical Committee of the AmsterdamUMC (Waiver: W-19_480 # 19.554). Informed consent of participants was waived by the Medical Ethical Committee.

### Data collection

Patients were included on workdays between 10 a.m. and 6 p.m. as at this time a researcher was available, and most patients were presented by ambulance. EMS diagnosis and patient characteristics such as age and sex were included. Also, years of working experience of the EMS nurses was collected. ED discharge diagnosis, the need for hospital admission and ICU admission was obtained from the electronic patient record.

### Data availability

Data is not publicly available. However, data is available upon reasonable request.

### Diagnostic agreement

Based on the International Classification of Diseases, 11th Revision, the preliminary and ED discharge diagnosis was grouped. Two researchers (PG and LV) grouped the diagnosis based on similarities in terms of symptoms and treatment with subsequent consensuses. In case more than one diagnosis was reported, the principal diagnosis was used. If a symptom was reported instead of a preliminary diagnosis, we classified this symptom into the most appropriate disease category, see Table 1.

After classification of the EMS and ED diagnosis, they were compared and listed as either concordant or discordant. Similar diagnosis included diagnosis that were either identical diagnosis or more precise diagnosis, i.e., bacterial pneumonia instead of pneumonia. Dissimilar diagnosis was those diagnosis that did not fulfil correct diagnosis criteria. Cohen’s kappa (κ) was used to measure this interrater reliability, interpreting 0.81-1.00 as almost perfect [9]. 

**Table 1:** Classification of diseases based on the ICD-11 codes

ICD-11 code	Classification
01	Infectious diseases
02–04	Emergencies blood, immune system, neoplasms
05	Endocrine or metabolic emergencies
06, 22	Mental and drug related emergencies
08–09	Neurological emergencies
11	Cardiovascular emergencies
12	Respiratory emergencies
13	Gastrointestinal emergencies
16	Genitourinary emergencies
15, 22	Traumatic injuries
21, 24	Acute internal emergencies

ICD-11 = international classification of diseases, 11th revision

### Outcomes

The primary outcome of this study was the rate of agreement between preliminary diagnoses made by ambulance staff and physician’s discharge diagnosis made in the ED, expressed as a percentage.

Secondary outcome included the association with concordant or discordant diagnosis with the need for hospital admission, ICU admission within 72 h and 28-day mortality.

### Statistical analysis

All analyses were performed using SPSS Statistics, version 27.0.0 (IBM Corporation, Armonk, NY, USA). Descriptive data were generated for all variables with frequencies, percentages, and mean [Standard deviation (SD)] or median [Interquartile range (IQR)] depending on the distribution of the data. Cases with a missing preliminary or discharge diagnosis were excluded. A p-value < 0.05 was considered statistically significant.

To compare accuracies between disease categories, a forest plot was constructed via Excel (Microsoft Excel, 2021), using the observed percentages and its 95%-confidence intervals for the different disease categories.

## Results

### Characteristics of study population

During inclusion period, a total of 800 patients were screened for eligibility criteria. Of these patients 65 were excluded due to missing diagnosis. This resulted in a study population of 735 patients. The median age was 66 years and 404 (55.0%) were male. Most of the preliminary diagnoses were neurological emergencies (24.4%) and cardiovascular emergencies (17.8%), see Table 2.

### Primary outcome

Out of the 735 patients included in the study, the preliminary diagnosis was accurate in 584 cases (79.5%). The interrater reliability agreement was almost perfect with a Cohen’s kappa value of 0.95. There was a difference in frequency between disease categories when comparing the population with a concordant preliminary diagnosis and the group with a discordant preliminary diagnosis, see Table 2. For instance, acute internal emergencies were one of the less frequent disease categories (4.6%) in patients with a concordant preliminary diagnosis but represented the largest percentage (23.8%) in the population with a discordant preliminary diagnosis.

**Table 2:** Characteristics total study population (*n* = 735), population with a concordant preliminary diagnosis (*n* = 584), and population with an discordant preliminary diagnosis (*n* = 151)

	Total study population (*n* = 735)	Concordant preliminary diagnosis (*n* = 584)	Discordant preliminary diagnosis (*n* = 151)	*P*-value
Sex, male	404 (55.0%)	314 (53.8%)	90 (59.6%)	0.199
Age (years), median [IQR]	66.0 [51.0–77.0]	65.0 [49.0–76.0]	69.0 [58.0–78.0]	0.179
Residence, home	670 (91.2%)	538 (92.1%)	132 (87.4%)	0.072
Pre-alerted patients by ambulance	524 (71.3%)	413 (70.7%)	111 (73.5%)	0.499
Doctor involved prehospital	359 (48.8%)	267 (45.7%)	92 (60.9%)	0.001
Length working experience EMS nurse (years), median [IQR]	10.0 [4.0-19.5]	10.0 [4.0–19.0]	11.0 [4.0–20.0]	0.294
Disease categories preliminary diagnosis EMS				
Acute internal emergencies	63 (8.6%)	27 (4.6%)	36 (23.8%)	< 0.001
Infectious diseases	113 (15.4%)	96 (16.4%)	17 (11.3%)	0.118
Emergencies blood, immune mechanism, neoplasms	17 (2.3%)	12 (2.1%)	5 (3.3%)	0.364
Endocrine and metabolic emergencies	23 (3.1%)	11 (1.9%)	12 (7.9%)	< 0.001
Mental and drug related emergencies	35 (4.8%)	26 (4.5%)	9 (6.0%)	0.440
Neurological emergencies	179 (24.4%)	162 (27.7%)	17 (11.3%)	< 0.001
Cardiovascular emergencies	131 (17.8%)	100 (17.1%)	31 (20.5%)	0.330
Respiratory emergencies	9 (1.2%)	8 (1.4%)	1 (0.7%)	0.491
Gastrointestinal emergencies	36 (4.9%)	25 (4.3%)	11 (7.3%)	0.132
Genitourinary emergencies	11 (1.5%)	6 (1.0%)	5 (3.3%)	0.051
Traumatic injuries	118 (16.0%)	111 (19.0%)	7 (4.6%)	< 0.001

N = number, IQR = inter-quartile range, ED = emergency department, EMS = Emergency medical services

### Differences in accuracy between disease categories

**Fig. 1:**
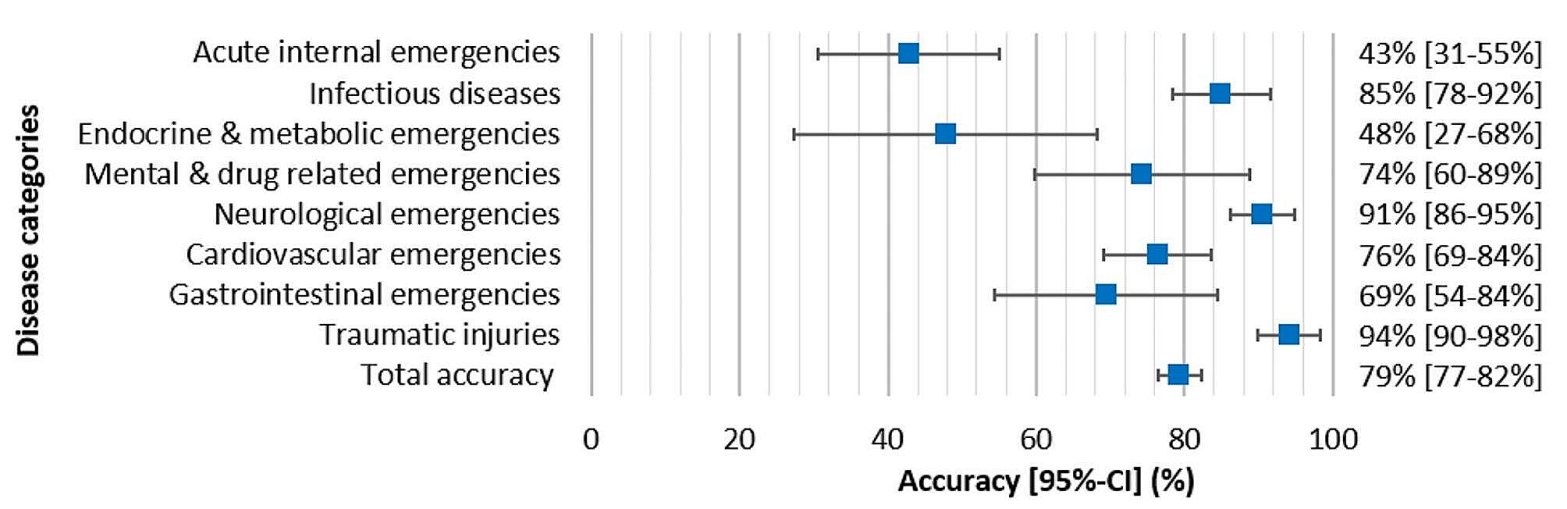
Forest plot. The vertical axis shows the disease categories with below the total accuracy. The horizontal axis represents the observed accuracy (box) with its 95%-confidence interval (horizontal line), both expressed in percentages. CI = confidence interval

As shown in Fig. 1, the highest accuracy of 94% was observed in traumatic injuries, and the lowest in acute internal emergencies (43%). The total accuracy of 79% is also represented with its 95%-confidence interval [77–82%]. For example, infectious diseases show a significant higher accuracy (85% [95%-CI: 78–92%]) compared to endocrine and metabolic emergencies (48% [95%-CI: 27–68%]).

### Secondary outcome

For 706 patients, 28-day mortality data was available. The 28-day mortality was not significantly different between patients with concordant diagnosis (6.6%) versus discordant diagnosis (8.5%), *p* = 0.429.

For 732 patients the need for ICU admission within 72 h after ED presentation was available. The need for ICU admission was not significantly different between the groups, 7.9% for concordant diagnosis versus 4.6% for discordant diagnosis, *p* = 0.171.

For all 735 patients the need for hospital admission after ED presentation was available. For patients with concordant preliminary diagnosis, 46.9% were admitted and for those with a discordant diagnosis this was 40.4%, *p* = 0.152.

## Discussion

This is the first Dutch study that examined to what extent the preliminary diagnosis made by the EMS is in agreement with ED discharge diagnosis. Based on these findings, we conclude that the accuracy of EMS nurses is high for patients with neurological emergencies, traumatic injuries, and those with infectious diseases. As hypothesized, the extent of agreement was higher compared to prior studies, however, the difference was only slightly increased. [5, 6] A possible explanation of this level of agreement, is the high level of education and traineeship for the Dutch EMS, however, another explanation is that many of the EMS nurses were previously working at the ED/ICU and therefore have a significant clinical experience.

On the contrary, a German study, examining diagnostic agreement for EMS emergency physicians, showed a slightly lower level of agreement (64%,) despite their high level of education [10]. This difference could be attributed to the use of a more specific disease classification (e.g., hypoglycaemia, hypertensive crisis), resulting in a stricter assessment of whether the EMS diagnosis was correct or not. In our perspective, a less detailed classification of the EMS diagnosis is more clinically relevant as this will engage the correct medical expertise (i.e., internal medicine, trauma, cardiac etc.) upon entry into the ED.

Furthermore, the variation in accuracies between the different disease categories are in concordance with the study of Koivulahti et al. [5] They stated that high accuracies are found in categories with specific observations, such as neurological failure, detectable injuries, and electrocardiographic abnormalities. The lower accuracies for endocrine and metabolic and acute internal emergencies might be attributed to the lack of readily available diagnostics and specific symptoms and the requirement for more profound knowledge of diseases including more extensive anamnesis and physical examination.

Particularly interesting is the high level of diagnostic agreement for patients with infectious diseases. A previous study in the Netherlands showed that in only 18% of (severe) sepsis, the disease severity was accurately diagnosed despite correct identification of an infectious disease state by the EMS. [11] This maybe be an interesting field of not only science to enhance the care for this specific sick group of patients.

### Strengths and limitations

Despite the prospectively collected data in two academic teaching hospitals, this study has several limitations. A limitation pertains to the relatively small sample size compared to the number of disease categories. Therefore, it is expected that the accuracy in these categories may be overoptimistic. [12] Considering the setting of this study (two university hospitals) may also influence external validity of this study as more peripherally located hospital may see different groups of patients with different diagnosis.

Also, patients were only included during working days between 10 a.m. and 6 p.m., while previous studies showed that the accuracy in overall clinical judgement fluctuates during the day and especially in the night. [13, 14] However, we can’t be certain at this point to what degree the overall (24/7) accuracy of diagnosis of EMS versus ED in the Netherlands may be. In addition, the ED physician’s discharge diagnosis was considered the gold standard for determining whether the preliminary diagnosis was correct or not. However, previous studies state that physicians can also make wrong conclusions. [15]

## Conclusion

In the Netherlands, the extent of agreement between EMS diagnosis and ED discharge diagnosis varies between categories. Accuracy is high in diseases with specific observations, e.g., neurological failure, detectable injuries, and electrocardiographic abnormalities. Further studies should investigate how we can use this to reduce time spend at the ED or to improve patient outcome.

## Data Availability

Data is not publicly available. However, data is available upon reasonable request. Please contact L.I.Veldhuis@amsterdamumc.nl for data requests.

## Abbreviations

ED: Emergency department
EMS: Emergency medical services
ICU: Intensive care unit
